# MicroRNA-181a Targets GNAI2 and Affects the Proliferation and Induction Ability of Dermal Papilla Cells: The Potential Involvement of the Wnt/β-Catenin Signaling Pathway

**DOI:** 10.3390/ijms25147950

**Published:** 2024-07-20

**Authors:** Mingliang He, Xiaoyang Lv, Joram M. Mwacharo, Yutao Li, Shanhe Wang, Wei Sun

**Affiliations:** 1 College of Animal Science and Technology, Yangzhou University, Yangzhou 225009, China; 13013706620@163.com; 2 Joint International Research Laboratory of Agriculture and Agri-Product Safety of Ministry of Education of China, Yangzhou University, Yangzhou 225009, China; 3 International Joint Research Laboratory in Universities of Jiangsu Province of China for Domestic Animal Germplasm Resources and Genetic Improvement, Yangzhou University, Yangzhou 225009, China; 4 International Centre for Agricultural Research in the Dry Areas, Addis Ababa 999047, Ethiopia; 5 CSIRO Agriculture and Food, 306 Carmody Rd, St. Lucia, Brisbane, QLD 4067, Australia

**Keywords:** miR-181a, GNAI2, DPCs, proliferation, induction ability, Wnt/β-catenin

## Abstract

Wool is generated by hair follicles (HFs), which are crucial in defining the length, diameter, and morphology of wool fibers. However, the regulatory mechanism of HF growth and development remains largely unknown. Dermal papilla cells (DPCs) are a specialized cell type within HFs that play a crucial role in governing the growth and development of HFs. This study aims to investigate the proliferation and induction ability of ovine DPCs to enhance our understanding of the potential regulatory mechanisms underlying ovine HF growth and development. Previous research has demonstrated that microRNA-181a (miR-181a) was differentially expressed in skin tissues with different wool phenotypes, which indicated that miR-181a might play a crucial role in wool morphogenesis. In this study, we revealed that miR-181a inhibited the proliferation and induction ability of ovine DPCs by quantitative Real-time PCR (qRT-PCR), cell counting Kit-8 (CCK-8), 5-ethynyl-2′-deoxyuridine (EdU), flow cytometry, and alkaline phosphatase staining. Then, we also confirmed G protein subunit alpha i2 (*GNAI2*) is a target gene of miR-181a by dual luciferase reporter assay, qRT-PCR, and Western blot, and that it could promote the proliferation and induction ability of ovine DPCs. In addition, *GNAI2* could also activate the Wnt/β-Catenin signaling pathway in ovine DPCs. This study showed that miR-181a can inhibit the proliferation and induction ability of ovine DPCs by targeting *GNAI2* through the Wnt/β-Catenin signaling pathway.

## 1. Introduction

The Hu sheep is a characteristic Chinese sheep breed known for its early sexual maturity, high fertility, abundant lambing, and excellent white patterned wool. Hu sheep are especially renowned for their ability to produce a white, wavy pattern of lamb skin, which is mainly formed by the bending of the wool [1]. As we know, wool is the raw material for many clothing products, so wool production is also an important source of economic income [2]. Wool is controlled by the wool follicle, which is an appendage of the sheep’s skin and can influence the length and diameter of the wool fiber [3]. Therefore, the growth and development of wool follicles are closely linked to the production of wool. Hair follicles (HFs) contain a variety of cells, including dermal papilla cells, hair stromal cells, outer-sheath root cells, and inner-sheath root cells, and the combined growth and interaction of these cells influence the length and diameter of the wool fiber [4]. Dermal papilla cells (DPCs) are an important cell type in hair follicles that can provide signaling and nutrients for hair-follicle growth processes [5]. It has also been reported that the number of DPCs is related to the diameter, crimp, and density of wool fibers [6]. Consequently, understanding the growth mechanism of DPCs is crucial for the analysis of hair follicle growth and development, thus contributing to a comprehensive understanding of wool fiber growth.

MicroRNAs (miRNAs) are a class of endogenous non-coding single-stranded RNA with a length of about 21–26 nucleotides and are encoded by about 3% of mammalian genes [7,8]. MiRNAs can regulate about 30% of protein-coding genes by targeting multiple genes and play a crucial role in regulating essential biological processes such as growth, development, and metabolism [8]. The increasing focus on miRNA function has also revealed its significant role in hair-follicle growth and development. MiR-218-5p within exosomes derived from DPCs was found to upregulate the expression of β-catenin, thereby promoting the growth of hair follicles in mice [9]. Similarly, the DPC exosome miRNA-181a-5p has been reported to activate the Wnt/β-catenin signaling pathway in hair-follicle stem cells by targeting the Wnt inhibitor *WIF1*, thereby promoting the growth and development of hair follicles in rabbits [10]. These studies demonstrate the regulatory function of miRNAs in hair-follicle growth and development and reflect the important role of DPCs in hair-follicle growth and development. Therefore, some miRNAs have also been found to be involved in the regulation of hair follicle growth in sheep and goats. For example, miRNA-203 can regulate the development of hair follicles of cashmere goats by downregulating the expression of *DDOST* and *NAE1* through specific binding to the 3′UTR region of *DDOST* and *NAE1* [11]. Similarly, miR-23b and miR-133 were found to jointly target *TGFβ2* and *NOTCH1* genes in skin fibroblasts to regulate skin fibroblast proliferation, apoptosis, and proliferation, thereby affecting hair follicle development in Subo merino sheep [12]. In a previous study, we found that miR-118a was differentially expressed in the skin tissues of different wool phenotypes. This finding suggests that miR-181a may play a crucial role in wool morphogenesis and the growth and development of wool hair follicles [13]. Numerous studies have shown that miRNAs play a vital role in the growth and development of hair follicles. They help to clarify the growth mechanism of wool by understanding the role of miRNAs in hair-follicle growth and development. Therefore, it is essential to investigate the function of miR-181a to elucidate the growth and development of Hu sheep wool follicles.

Normally, miRNAs play a key role in regulating gene expression by binding to target mRNA [14,15]. Here, we identified the G protein subunit alpha i2 (*GNAI2*) as a potential target gene of miR-181a. The *GNAI2* is a subtype of the guanine nucleotide-binding proteins (*GNAS*) located in the cytoplasm. *GNAS* can activate adenylate cyclase enzymes, which leads to the production of cyclic adenosine monophosphate (cAMP). This activation can facilitate the opening of ion channels and regulate the transmission of hormones and neurotransmitters, cell proliferation, apoptosis, and migration [16,17]. Research has demonstrated that *GNAI2* plays a role in promoting the proliferation and inhibiting the apoptosis of rabbit melanocytes [18]. Furthermore, *GNAI2* has been found to facilitate the proliferation of ovarian cancer cells (EOCs) through involvement in the miR-222-3p/*GNAI2*/AKT pathway [19]. The function of *GNAI2* in sheep has been studied, with findings indicating its involvement in the proliferation and differentiation of skeletal muscle cells [20]. Furthermore, *GNAI2* has been identified as a target gene regulated by miR-193b, affecting hair color formation in cashmere goats [21]. Nevertheless, the specific role of *GNAI2* in sheep hair follicles remains unexplored.

In this study, we aimed to study the effects of miR-181a on the proliferation and induction ability of sheep DPCs and explore the mechanism of miR-181a regulating the proliferation and induction ability of sheep DPCs. This study will help to elucidate the influence mechanism of non-coding RNA on ovine DPC characteristics and wool-follicle growth and development of Hu sheep.

## 2. Results

### 2.1. MiR-181a Inhibits the Proliferation of Ovine DPCs

**Figure 1:**
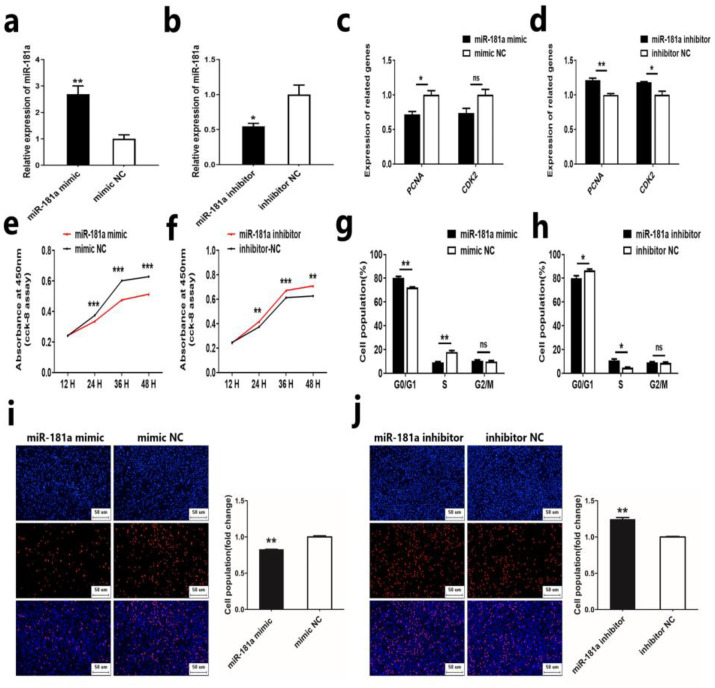
MiR-181a inhibits the proliferation of ovine DPCs. (**a**,**b**) Expression of miR-181a after transfection of ovine DPCs with miR-181a mimic and miR-181a inhibitor. (**c**,**d**) Expression of *PCNA* and *CDK2* after transfection of sheep DPCs with miR-181a mimic and miR-181a inhibitor, respectively. (**e**,**f**) CCK-8 assay after transfection of ovine DPCs with miR-181a mimic and miR-181a inhibitor, respectively. (**g**,**h**) Cell cycle assay after transfection of sheep DPCs with miR-181a mimic or miR-181a inhibitor. (**i**,**j**) EdU assay after transfection of sheep DPCs with miR-181a mimic or miR-181a inhibitor; the scale is 100 µm. The unpaired Student’s *t*-test was used for statistical significance (^ns^ *p* > 0.05; * *p* < 0.05; ** *p* < 0.01; *** *p* < 0.001).

In a previous study, we discovered that miR-181a was differentially expressed in different wool phenotype skin tissues, which led us to hypothesize that miR-181a may play a crucial role in wool morphogenesis [13]. Given the important role of DPCs in HFs, we aimed to understand the potential role of miR-181a in the growth and development of HF and wool growth by investigating the effect of miR-181a on ovine DPCs. First, we investigated the effect of miR-181a on ovine DPC using a mimic and inhibitor of miR-181a. Quantitative Real-time PCR (qRT-PCR) assay showed that miR-181a mimic could significantly increase the expression of miR-181a in ovine DPCs, while miR-181a inhibitor has the opposite effect (Figure 1a,b). Next, we transfected miR-181a mimic and inhibitor into ovine DPCs and detected the proliferation of ovine DPCs by qRT-PCR, cell counting Kit-8 (CCK-8), 5-ethynyl-2′-deoxyuridine (EdU), and flow cytometry. qRT-PCR analysis demonstrated that overexpression of miR-181a could reduce the mRNA expression of *PCNA* and *CDK2*, while interference of miR-181a could enhance the mRNA expression of *PCNA* and *CDK2* (Figure 1c,d). In addition, CCK-8 and EdU assays showed that overexpression of miR-181a could suppress the viability and proliferation of ovine DPCs (Figure 1e,i). Conversely, the knockdown of miR-181a could promote the viability and proliferation of ovine DPCs (Figure 1f,j). In addition, the cell cycle assay showed that miR-181a could regulate the proliferation of ovine DPCs by modulating the G0/G1 phase and S phase (Figure 1g,h). These results indicated that miR-181a could inhibit the proliferation of ovine DPCs.

### 2.2. MiR-181a Inhibits the Induction Ability of Ovine DPCs

**Figure 2:**
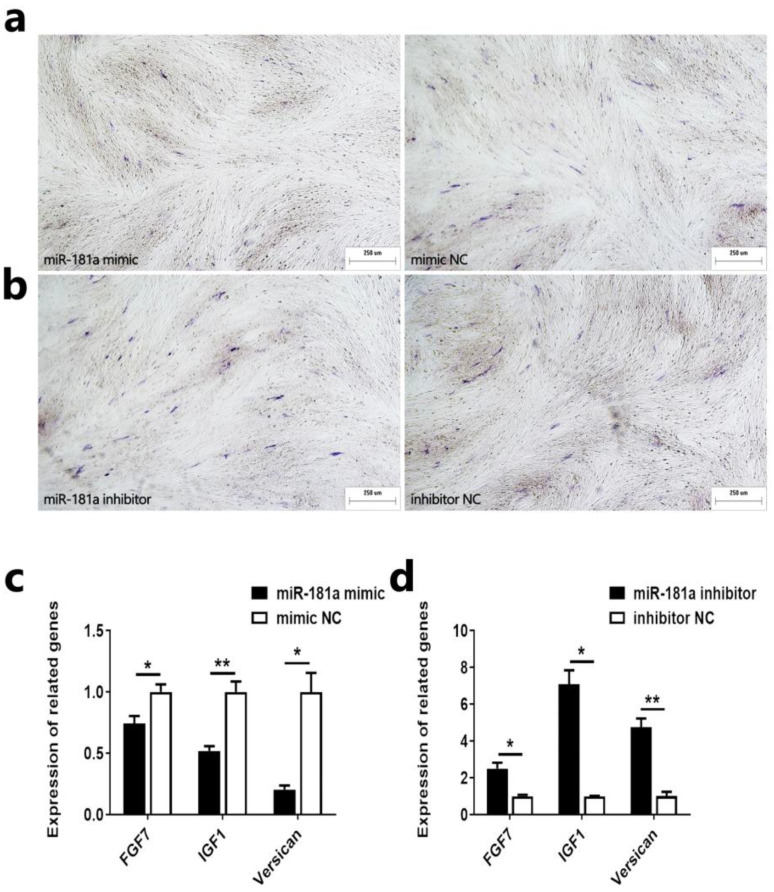
MiR-181a inhibits the induction ability of ovine DPCs. (**a**,**b**) Alkaline phosphatase staining after transfection of ovine DPCs with miR-181a mimic or miR-181a inhibitor; the scale is 250 µm. (**c**,**d**) Expression of *FGF7*, *IGF1*, and *Versican* after transfection of ovine DPCs with miR-181a mimic and miR-181a inhibitor. The unpaired Student’s *t*-test was used for statistical significance (* *p* < 0.05; ** *p* < 0.01).

DPCs have maintenance and induction effects, characterized by agglutinative growth behavior in vitro and the ability to induce the formation of new hair follicles [5]. To investigate the effect of miR-181a on the induction ability of ovine DPCs, we first determined the alkaline phosphatase activity of ovine DPCs after the overexpression or knockdown of miR-181a in ovine DPCs. The result showed that the miR-181a mimic could inhibit the alkaline phosphatase activity of ovine DPCs (Figure 2a). The miR-181a inhibitor could promote the alkaline phosphatase activity of ovine DPCs (Figure 2b). Next, we examined the expression of inducible ability-related genes (*FGF7*, *IGF1*, and *Versican*) after overexpression or knockdown of miR-181a in ovine DPCs [22,23]. qRT-PCR assays showed that the miR-181a mimic could inhibit the mRNA expression of *FGF7*, *IGF1*, and *Versican* (Figure 2c). And the miR-181a inhibitor could promote the mRNA expression of *FGF7*, *IGF1*, and *Versican* (Figure 2d). These results suggest that miR-181a can inhibit the induction ability of ovine DPCs.

### 2.3. GNAI2 Is a Target Gene of miR-181a

**Figure 3:**
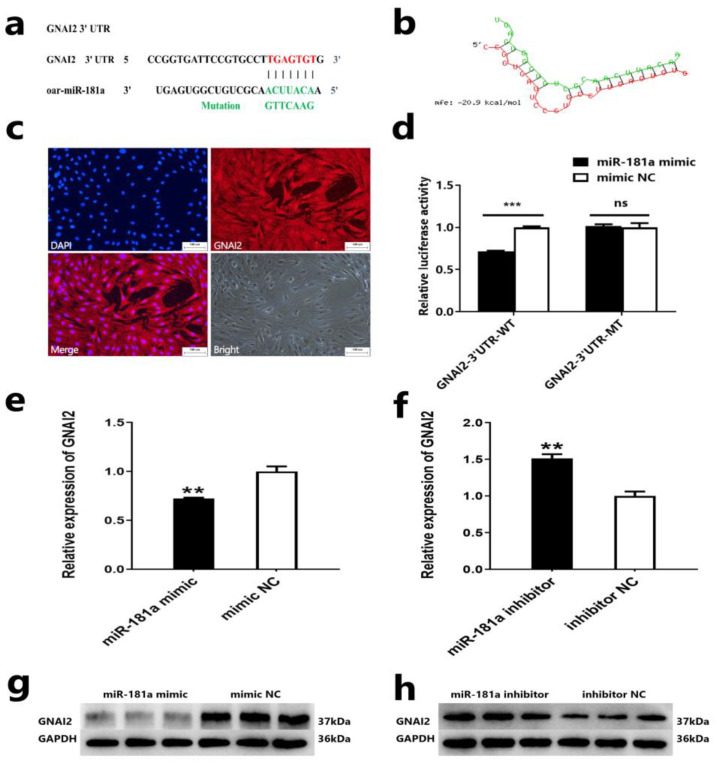
*GNAI2* is a target gene of miR-181a. (**a**) The potential binding site of the miR-181a seed sequence in the 3′UTR region of the *GNAI2* gene. The seed sequences of miR-181a are highlighted in red, and the wild-type and mutant sequences of the 3′UTR region of the *GNAI2* gene are highlighted in green. (**b**) The interaction model between miR-181a and the 3′UTR of *GNAI2* was analyzed by RNAhybrid. (**c**) *GNAI2* is expressed in Hu sheep DPCs; the scale is 50 µm. (**d**) The luciferase assays after transfection of the vectors PMIR-GNAI2-3′UTR-WT or PMIR-GNAI2-3′UTR-MT and miR-181a mimic or mimic-NC into HEK293T cells. (**e**,**f**) The mRNA expression level of *GNAI2* after overexpression or knockdown of miR-181a in ovine DPCs. (**g**,**h**) The protein expression of *GNAI2* after overexpression or knockdown of miR-181a in ovine DPCs. The unpaired Student’s *t*-test was used for statistical significance (^ns^ *p* > 0.05; ** *p* < 0.01; *** *p* < 0.001).

To better understand the molecular regulatory mechanism of miR-181a in the proliferation and induction ability of ovine DPCs, we predicted the target gene of miR-18a. The results showed that the miR-181a seed sequence could bind to the 3′UTR region of the *GNAI2* gene, and the interaction model analysis of RNAhybrid again showed that the binding between miR-181a and *GNAI2* was stable (Figure 3a,b). These results suggested that the *GNAI2* gene may be a potential target gene of miR-181a. Next, we identified the expression of the *GNAI2* gene in Hu sheep DPC using immunofluorescence staining (Figure 3c). Then, we verified whether the *GNAI2* gene is the target gene of miR-181a by performing a dual luciferase reporter assay. We transfected the vectors PMIR-GNAI2-3′UTR-WT or PMIR-GNAI2-3′UTR-MT and miR-181a mimic or mimic-NC into HEK293T cells. The dual luciferase reporter assay showed that the luciferase activity of HEK293T cells was significantly reduced after transfer with miR-181a mimic and PMIR-GNAI2-3′UTR-WT vector compared to transfer with the mimic-NC and the PMIR-GNAI2-3′UTR-WT vector; the luciferase activity of HEK293T cells was not different after transfection with the miR-181a mimic and the PMIR-GNAI2-3′UTR-MT vector compared to transfection with the mimic-NC and the PMIR-GNAI2-3′UTR-MT vector (Figure 3d). In addition, we also detected mRNA and protein expression after overexpression or knockdown of miR-181a in ovine DPCs. qRT-PCR and Western blot assay showed that overexpression of miR-181a could inhibit mRNA and protein expression of *GNAI2*, while interference of miR-181a resulted in an opposite effect (Figure 3e–h). These results indicated that *GNAI2* is a target gene of miR-181a.

### 2.4. GNAI2 Promotes the Proliferation of Ovine DPCs

**Figure 4:**
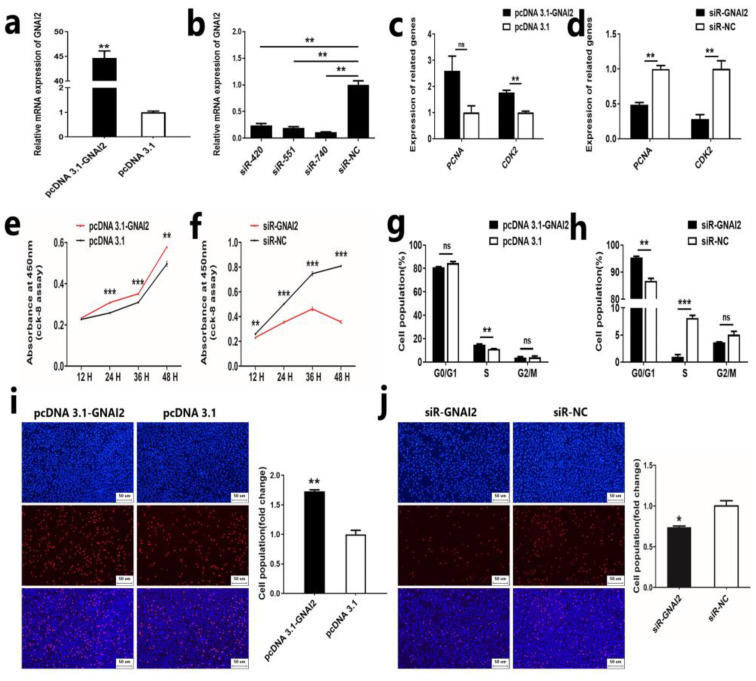
*GNAI2* promotes the proliferation of ovine DPCs. (**a**,**b**) Expression of *GNAI2* after overexpression or knockdown of *GNAI2* in ovine DPCs. (**c**,**d**) Expression of *PCNA* and *CDK2* after overexpression or knockdown of *GNAI2* in ovine DPCs. (**e**,**f**) CCK-8 assay after overexpression or knockdown of *GNAI2* in ovine DPCs. (**g**,**h**) Cell cycle assay after overexpression or knockdown of *GNAI2* in ovine DPCs. (**i**,**j**) EdU assay after overexpression or knockdown of *GNAI2* in ovine DPCs; the scale is 100 µm. The unpaired Student’s *t*-test was used for statistical significance (^ns^ *p* > 0.05; * *p* < 0.05; ** *p* < 0.01; *** *p* < 0.001).

Based on the results of target gene identification of miR-181a, we further investigated the effect of the target gene *GNAI2* on the proliferation of ovine DPCs. First, we constructed the overexpression vector and designed the siRNA sequence of *GNAI2*. qRT-PCR assay showed that the mRNA expression level of *GNAI2* was increased after overexpression of *GNAI2*, while the mRNA expression level of *GNAI2* was reduced after knockdown of *GNAI2* (Figure 4a,b). These results indicated that the overexpression vector and siRNA sequence could be used for the subsequent experiments. Next, we transfected the *GNAI2* overexpression vector and siRNA into ovine DPCs and detected the proliferation of ovine DPCs by qRT-PCR, CCK-8, EdU, and flow cytometry. qRT-PCR analysis showed that overexpression of *GNAI2* could enhance the mRNA expression of *PCNA* and *CDK2*, while interference of *GNAI2* could reduce the mRNA expression of *PCNA* and *CDK2* (Figure 4c,d). In addition, CCK-8 and EdU assays showed that overexpression of *GNAI2* could promote the viability and proliferation of ovine DPCs, respectively (Figure 4e,i). Conversely, knockdown of *GNAI2* could suppress the viability and proliferation of ovine DPCs (Figure 4f,j). In addition, the cell cycle assay showed that *GNAI2* could regulate the proliferation of ovine DPCs by modulating the G0/G1 phase and S phase (Figure 4g,h). These results suggested that *GNAI2* could promote the proliferation of ovine DPCs.

### 2.5. GNAI2 Promotes the Induction Ability of Ovine DPCs

**Figure 5:**
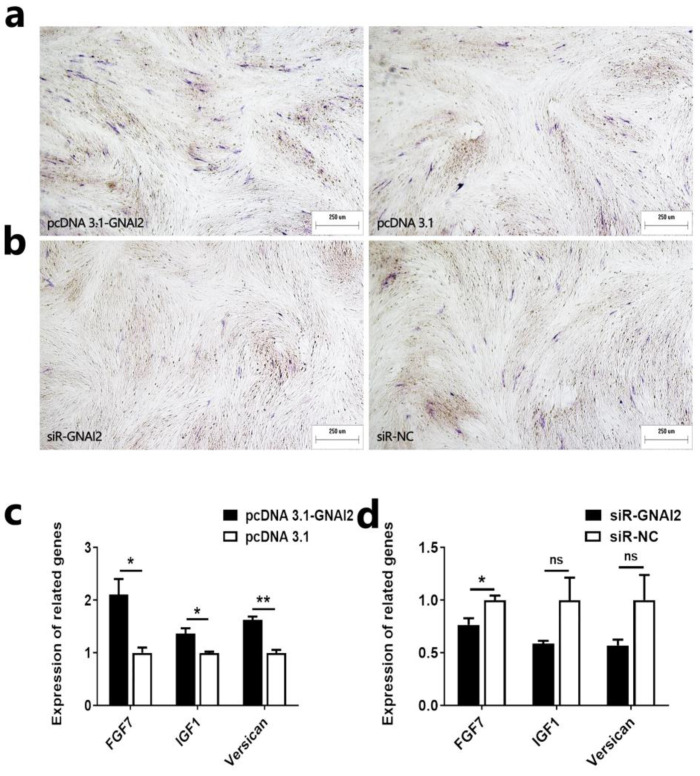
*GNAI2* promotes the induction ability of ovine DPCs. (**a**,**b**) Alkaline phosphatase staining after overexpression or knockdown of *GNAI2* in ovine DPCs; the scale is 250 µm. (**c**,**d**) Expression of *FGF7*, *IGF1*, and *Versican* after overexpression or knockdown of *GNAI2* in ovine DPCs. The unpaired Student’s *t*-test was used for statistical significance (^ns^ *p* > 0.05; * *p* < 0.05; ** *p* < 0.01).

We have also investigated the effect of *GNAI2* on the induction ability of ovine DPCs. First, we determined the alkaline phosphatase activity of ovine DPCs after overexpression or knockdown of *GNAI2* in ovine DPCs. The result showed that overexpression of *GNAI2* could promote the alkaline phosphatase activity of ovine DPCs (Figure 5a). Knockdown of *GNAI2* could inhibit the alkaline phosphatase activity of ovine DPCs (Figure 5b). Next, we examined the expression of inducible ability-related genes after overexpression or knockdown of *GNAI2* in ovine DPCs. qRT-PCR assay showed that overexpression of *GNAI2* could promote the mRNA expression of *FGF7*, *IGF1*, and *Versican* (Figure 5c). Suppression of *GNAI2* could inhibit the mRNA expression of *FGF7*, *IGF1*, and *Versican* (Figure 5d). These results suggest that *GNAI2* can promote the induction ability of ovine DPCs.

### 2.6. GNAI2 Is Involved in the Wnt/β-Catenin Signaling Pathway of Ovine DPCs

**Figure 6:**
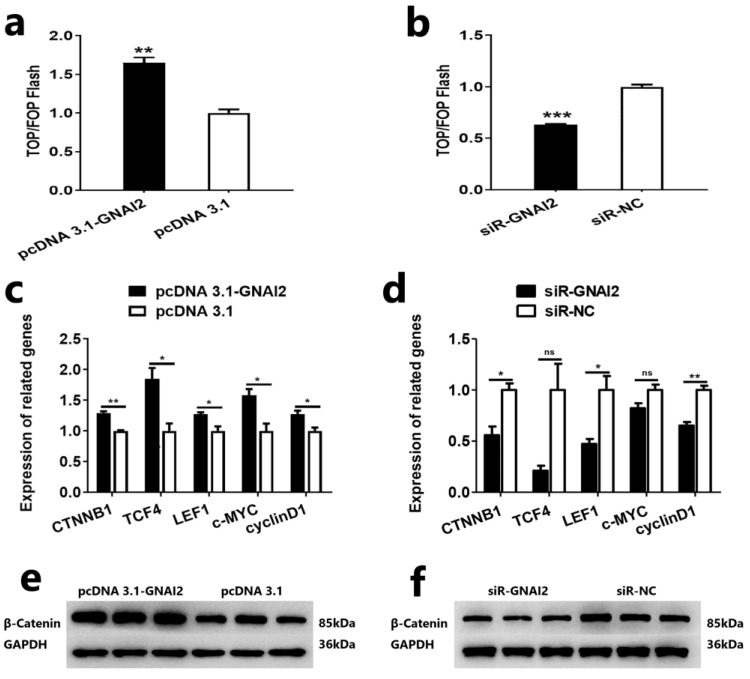
*GNAI2* is involved in the Wnt/β-catenin signaling pathway of ovine DPCs. (**a**,**b**) TOP/FOP flash assays after overexpression or knockdown of *GNAI2* in ovine DPCs. (**c**,**d**) Expression of *CTNNB1*, *TCF4*, *LEF1*, *c-MYC*, and *cyclinD1* after overexpression or knockdown of *GNAI2* in ovine DPCs. (**e**,**f**) Expression of β-catenin after overexpression or knockdown of *GNAI2* in ovine DPCs. The unpaired Student’s *t*-test was used for statistical significance (^ns^ *p* > 0.05; * *p* < 0.05; ** *p* < 0.01; *** *p* < 0.001).

The Wnt/β-catenin signaling pathway plays an important role in ovine DPCs [24]. In this study, we also investigated the effect of *GNAI2* on the Wnt/β-catenin signaling pathway and gained a preliminary understanding of whether *GNAI2* regulated the proliferation and induction ability of ovine DPCs by activating the Wnt/β-catenin signaling pathway. First, we measured the effect of *GNAI2* on the transcriptional activity of β-catenin/TCF in ovine DPCs. The results showed that overexpression of *GNAI2* could increase the activity of the Wnt/β-catenin signaling pathway in ovine DPCs and knockdown of *GNAI2* could decrease the activity of the Wnt/β-catenin signaling pathway in ovine DPCs (Figure 6a,b). Next, we detected the genes associated with the Wnt/β-catenin signaling pathway in ovine DPCs. qRT-PCR assay showed that *GNAI2* could increase the mRNA expression of *CTNNB1*, *TCF4*, *LEF1*, *c-MYC*, and *cyclinD1* (Figure 6c,d). Western blot assay showed that *GNAI2* could increase the protein expression of β-catenin (Figure 6e,f). These results suggested that *GNAI2* regulated the proliferation and induction ability of ovine DPCs via the Wnt/β-catenin signaling pathway.

## 3. Discussion

Hair follicles are tiny appendages of skin composed of epidermis and dermis and their growth and development determine the growth of hair [3,25]. Hair follicles contain various cells, including DPCs, hair matrix cells, inner root-sheath cells, outer root-sheath cells, etc. These cells can be specifically regulated, and can also affect the growth, development, and cycle of hair follicles through the interaction of cell proliferation and differentiation [26,27,28]. DPCs are the dermal part of the hair follicle, located at the base of the hair follicle and surrounded by hair matrix cells. DPCs are the signaling centers of hair follicles, which can affect the differentiation of hair matrix cells and hair growth through signaling [29,30]. Studies have shown that the number of DPCs in mouse hair follicles can affect the shape of mouse hair, and that reducing the number of DPCs in mouse hair follicles can transform the mouse “awl hairs” into “zigzag hairs” [31]. In addition, it has been found that the hair follicles cannot enter the growth phase, and the hair shape changes after using a laser to specifically eliminate the DPCs [32]. These studies have shown that hair follicle DPCs play an important role in the normal growth and development of hair follicles and hair morphogenesis, so understanding the mechanism of DPC growth is of great significance for analyzing the mechanism of hair-follicle growth and development and hair morphogenesis.

With the further study of miRNA function, the researchers found that hair-follicle growth and development are not only affected by genes and signaling pathways but also by the epigenetic regulatory factors miRNAs. High-throughput sequencing of cashmere goat skin tissue revealed 172 novel miRNAs and 399 known miRNAs in the skin tissue, of which 26, 41, and 55 miRNAs were only expressed during hair-follicle growth, degeneration, and quiescence [33]. By knocking out the *DicerI* gene related to miRNA maturation in mice, Yi et al. found that the proliferation rate of mouse hair-follicle cells decreased, the apoptosis rate accelerated, and the formation of hair follicles slowed down in the first week of birth. These results indicated that mature miRNA was involved in the formation of mouse hair follicles [34]. Our previous study found that miR-181a was differentially expressed in the skin of Hu sheep with different pattern types [13]. Combined with previous studies, we speculated that miR-181a plays an important role in hair-follicle growth and development and hair morphogenesis, and may play a corresponding role by influencing the number of DPCs. To understand the role of miR-181a in the growth of DPCs and the growth and development of hair follicles, we conducted a series of in vitro experiments and found that overexpression of miR-181a could inhibit the proliferation and induction of ovine DPCs. These results suggested that miR-181a may affect the growth and development of hair follicles by acting on ovine DPC and regulating its proliferation and induction ability.

A miRNA is usually able to target multiple target genes and affect the expression of the target genes [14,15,35]. Studies have shown that miR-181a-5p in the exosomes of rabbit hair follicle DPC could activate the Wnt/β-catenin signaling pathway by targeting the Wnt inhibitor WIF1 gene, thereby regulating hair follicle growth and development [10]. Here, the *GNAI2* gene was identified as a target gene of miR-181a by software prediction, qRT-PCR assay, Western blot assay, and dual-luciferase assay. These results indicated that miR-181a could regulate the proliferation and growth characteristics of DPCs and hair-follicle growth and development by targeting the *GNAI2* gene in Hu sheep hair-follicle DPCs. Previous studies have found that *GNAI2* was mainly expressed in mouse retinal endothelial cells, and overexpression of *GNAI2* in endothelial cells could induce proangiogenic activity and enhance cell proliferation, migration, invasion, and capillary tube formation [36]. In addition, *GNAI2* has been identified as a target gene of microRNA-138; its expression was regulated by microRNA-138 and it was involved in the cell cycle, cell proliferation, and apoptosis of tongue squamous cell carcinoma (TSCC) [37]. Here, We found that overexpression of *GNAI2* could promote the proliferation and induction ability of ovine DPCs, suggesting that *GNAI2* may affect the growth and development of hair follicles by regulating the proliferation and induction ability of ovine DPCs.

The Wnt/β-catenin signaling pathway plays an important role in the growth and development of hair follicles and hair growth [38]. The role of the Wnt/β-catenin signaling pathway in the proliferation of papilla cells of sheep hair follicles has also been extensively studied. *SOX18* has been reported to promote the proliferation of DPCs of Hu sheep hair follicles by activating the Wnt/β-Catenin signaling pathway [24]. Furthermore, the effects of the Wnt/β-Catenin signaling pathway on the induction ability of DPCs have also been reported. MiR-195-5p was found to affect the inductivity of hair follicle DPCs by inhibiting the activity of the Wnt/β-Catenin signaling pathway [39]. In this study, we found that *GNAI2* could enhance the activity of the Wnt/β-Catenin signaling pathway in ovine DPC and promote the expression of Wnt/β-Catenin signaling pathway-related genes and proteins. This finding suggested that *GNAI2* could promote the proliferation and induction ability of ovine DPCs by activating the Wnt/β-Catenin signaling pathway.

## 4. Materials and Methods

### 4.1. Animals and Ethics Statement

Suzhou Sheep Farm (Suzhou, China) provided Hu sheep skin for cell isolating. Skin samples were collected from a healthy 3-day-old Hu sheep lamb following the guidelines specified in the “Jiangsu Province Laboratory Animal Management Measures”. The Animal Ethics Committee of Yangzhou University (Approval number: No. 202103279) approved the animal experiment protocol.

### 4.2. Cell Isolation, Culture, and Transfection

DPCs were isolated from the growth-stage hair follicles of Hu sheep lamb-skin tissue. The cell isolation process is as follows. First, the skin tissue was cut into small pieces along the lengthwise direction of hair-follicle growth using a scalpel. Then, hair follicles were extracted from skin tissue, and their swollen ends were disrupted using tweezers. Next, the bulged ends of hair follicles were implanted into a 12-well cell culture dish (NEST Biotechnology, Wuxi, China) for cell migration and culture. DMEM-F12 (Sigma-Aldrich, St. Louis, MO, USA) medium was used for cell culture, and the medium was added to 10% fetal bovine serum (Gibco, Grand Island, NY, USA) and 1% penicillin-streptomycin-amphotericin (Solarbio, Beijing, China). The cell culture condition was 5% CO_2_ at 37 °C. The jetPRIME Transfection Reagent (Polyplus, Illkirch, France) was used for cell transfection.

### 4.3. Total RNA Extraction, cDNA Synthesis, Primer Design, and qRT-PCR

DPCs were seeded in a 12-well cell culture dish and the cell transfection density was approximately 50%. Trizol (Takara, Dalian, China) was used for the total RNA extraction. MiRNA 1st strand cDNA synthesis Kit (by stem-loop) (Vazyme, Nanjing, China) and one-step reverse transcription Kit (Tiagen, Beijing, China) were used for miRNA cDNA and gene cDNA synthesis, respectively. MiRNA Universal SYBR qPCR Master Mix (Vazyme, Nanjing, China) and 2×TSINGKE^®^ Master qPCR mix (Tsingke, Nanjing, China) were used for miR-181a expression level and gene expression level detection, respectively. *U6* and *GAPDH* served as the housekeeping genes. Each sample was tested three replicated times. The 2^−ΔΔCT^ method was used to calculate the relative expression of miR-181a and genes [40]. MiRNA Design V1.01 software (Vazyme, Nanjing, China) was used to design primers of miR-181a and *U6*. Premier Primer 5.0 software (Premier Bio-soft International, Palo Alto, CA, USA) was used to design gene primers. All primer sequences are provided in Table 1 and Table 2.

### 4.4. Plasmid Construction and RNA Oligonucleotides

PrimeSTAR^®^ Max DNA Polymerase (Takara, Dalian, China) was used to amplify the coding domain sequence (CDS) of the sheep *GNAI2* gene. Then, the restriction sites HindIII and BamHI were selected for double digestion of the pcDNA 3.1+ reporter vector, and the CDS fragment was inserted into the vector. Moreover, PrimeSTAR^®^ Max DNA Polymerase (Takara, Dalian, China) was also used to amplify the 3′ untranslated region (3′ UTR) region of the *GNAI2* gene, which contains the predicted binding site of miR-181a and *GNAI2*. The restriction sites HindIII and MIuI were selected for double digestion of the PMIR dual-luciferase reporter vector, and the 3′ UTR fragment was inserted into the vector. The Fast Mutagenesis Kit V2 (Vazyme, Nanjing, China) was used to construct the mutant-type dual-luciferase reporter plasmid. All these recombinant plasmids were sequenced to verify the successful construction. All primers used for vector construction are provided in Table 3.

The miR-181a mimic, mimic-NC (negative control), miR-181a inhibitor, inhibitor NC, and small interfering RNAs (siRNAs) to suppress the expression of *GNAI2* and the siRNA negative control (NC) were designed and synthesized by GenePharma (Suzhou, China). The oligonucleotide sequences of siRNA and NC are provided in Table 4.

### 4.5. CCK-8 Assay

DPCs were seeded into a 96-well cell culture dish and the cell transfection density was approximately 30%. We used the CCK-8 Kit (Vazyme, Nanjing, China) to detect cell viability at 12 h (after DPCs were transfected), 24 h, 36 h, and 48 h. A microplate reader (EnSpire, Perkin Elmer, Waltham, MA, USA) was used to detect the cell absorbance at 450 nm.

### 4.6. EdU Assay

DPCs were seeded into a 24-well cell culture dish and the cell transfection density was approximately 50%. First, DPCs were fixed for 30 min with 4% paraformaldehyde (Solarbio, Beijing, China). Second, DPCs were transparented using Triton X-100. Finally, DPCs were conducted by an EdU Apollo In Vitro Imaging Kit (RiboBio, Guangzhou, China). An inverted fluorescence microscope (Nikon, Tokyo, Japan) was used to observe and capture images of the stained DPCs and Image Pro Plus 6.0 software (Media Cybernetics, Rockville, MD, USA) was used to analyze images.

### 4.7. Cell Cycle Assay

DPCs were seeded into a 6-well cell culture dish and the cell transfection density was approximately 50%. First, DPCs were collected by trypsin (Solarbio, Beijing, China). Second, DPCs were fixed for 12 h with 70% ethanol. Finally, DPCs were stained with propidium iodide (50 µg/mL, Solarbio, Beijing, China) containing RNaseA (50 µg/mL, TianGen, Beijing, China) and incubated at 37 °C in darkness for 30 min. A FACSAria SORP flow cytometer (BD Company, Franklin, NJ, USA) was used for cell stage analysis. ModFit LT 5.0 software (Verity Software House, Bedford, MA, USA) was used for data analysis.

### 4.8. Alkaline Phosphatase Activity Assay

DPCs were seeded into a 6-well cell culture dish and the cell transfection density was approximately 50%. First, DPCs were fixed for 0.5 h with 4% formaldehyde (Solarbio, Beijing, China). Then, the alkaline phosphatase activity of DPC was detected by a BCIP/NBT Alkaline Phosphatase Color Development Kit (Beyotime, Shanghai, China). An inverted fluorescence microscope (Nikon, Tokyo, Japan) was used to observe and capture images of the stained DPCs.

### 4.9. Immunofluorescence Assay

DPCs were seeded into a 6-well cell culture dish and the cell transfection density was approximately 50%. First, DPCs were washed with 1X PBS (Solarbio, Beijing, China) and fixed with 4% paraformaldehyde (Solarbio, Beijing, China). Second, DPCs were permeated by 0.5% Triton X-100 (Solarbio, Beijing, China) and incubated with 5% BSA (Solarbio, Beijing, China). Finally, DPCs were incubated in darkness at 4 °C using the primary antibody. The next day, DPCs were washed with 1× PBST (Solarbio, Beijing, China) and incubated at 37 °C using the secondary antibody. DAPI (Beyotime, Shanghai, China) was used for the staining of cell nuclei. An inverted fluorescence microscope (Nikon, Tokyo, Japan) was used to observe and capture images of the stained DPCs. Antibodies and their respective dilution ratios are presented as follows. Primary antibodies: GNAI2 (Beyotime, Shanghai, China, 1:400). Secondary antibodies: Multi-rAb CoraLite^®^ Plus 594-Goat Anti-Mouse Recombinant Secondary Antibody (H+L) (proteintech, Wuhan, China, 1:400).

### 4.10. Dual-Luciferase Assay

HEK293T cells were seeded into a 24-well cell culture dish and the cell transfection density was approximately 50%. First, HEK293T cells were co-transfected using the wild-type vector (PMIR-GNAI2-3′UTR-WT) or the mutant vector (PMIR-GNAI2-3′UTR-MT) with miR-181a mimic or NC and the pRL-TK reporter vector, respectively. Then, a dual-luciferase detection kit (Vazyme, Nanjing, China) was used to process the HEK293T cells and a multi-mode micropore detection system (EnSpire, PerkinElmer, Waltham, MA, USA) was used to detect the luciferase activity.

### 4.11. TOP/FOP-Flash Wnt Report Assays

DPCs were seeded into a 24-well cell culture dish and the cell transfection density was approximately 50%. The TOP/FOP-flash plasmid (Beyotime, Shanghai, China) was used to estimate the transcription activity of β-catenin/TCF in DPCs. First, DPCs were co-transfected using pcDNA 3.1-GNAI2 or pcDNA 3.1 with the TOP or FOP-flash plasmid and the pRL-TK reporter vector, respectively. Then, a dual-luciferase detection kit (Vazyme, Nanjing, China) was used to process the DPCs and a multi-mode micropore detection system (EnSpire, PerkinElmer, Waltham, MA, USA) was used to detect the luciferase activity.

### 4.12. Total Protein Extraction, and Western Blot Assay

DPCs were seeded into a 6-well cell culture dish and the cell transfection density was approximately 50%. First, RIPA cell lysates (Beyotime, Shanghai, China) and protease inhibitors (Beyotime, Shanghai, China) were used to collect proteins. Second, the BCA Protein Quantification Kit (Vazyme, Nanjing, China) was used to determine protein concentration. Third, 10% polyacrylamide gel electrophoresis was used to obtain the target protein, and the target protein was transferred to the PVDF membrane (Solarbio, Beijing, China). Finally, the primary antibody was used to incubate the PVDF membrane at 4 °C in darkness for 12 h. The following day, the secondary antibody was used to incubate the PVDF membrane at 37 °C in darkness for 1 h. Electrochemiluminescence (ECL) (Beyotime, Shanghai, China) was used to display the imprinting of the PVDF membrane. A stripping buffer (Beyotime, Shanghai, China) was used to remove the primary and secondary antibodies after detecting GNAI2/GAPDH protein expression. Subsequently, the GAPDH/GNAI2 primary antibody and related secondary antibody were used to incubate the PVDF membrane for detecting GAPDH protein expression. The ChemDocTMTouch Imaging System (Bio-Rad, Hercules, CA, USA) was used to measure protein expression level. Antibodies and their respective dilution ratios are presented as follows. Primary antibodies: GNAI2 (Beyotime, Shanghai, China, 1:2000), β-catenin (Beyotime, Shanghai, China, 1:1000), GAPDH (proteintech, Wuhan, China, 1:5000). Secondary antibodies: HRP-conjugated Goat anti-Mouse IgG (H+L) (ABclonal, Wuhan, China, 1:5000).

### 4.13. Statistical Analysis

The SPSS 25.0 software (SPSS Inc., Chicago, IL, USA) was used to analyze statistics and the unpaired Student’s *t*-test was used to analyze the two-group data. Only when *p* < 0.05 (*), *p* < 0.01 (**), or *p* < 0.001 (***) were the data considered statistically significant. Each experiment group was tested three replicated times. All data are presented as means ± SEM (standard error of the mean).

## 5. Conclusions

**Figure 7:**
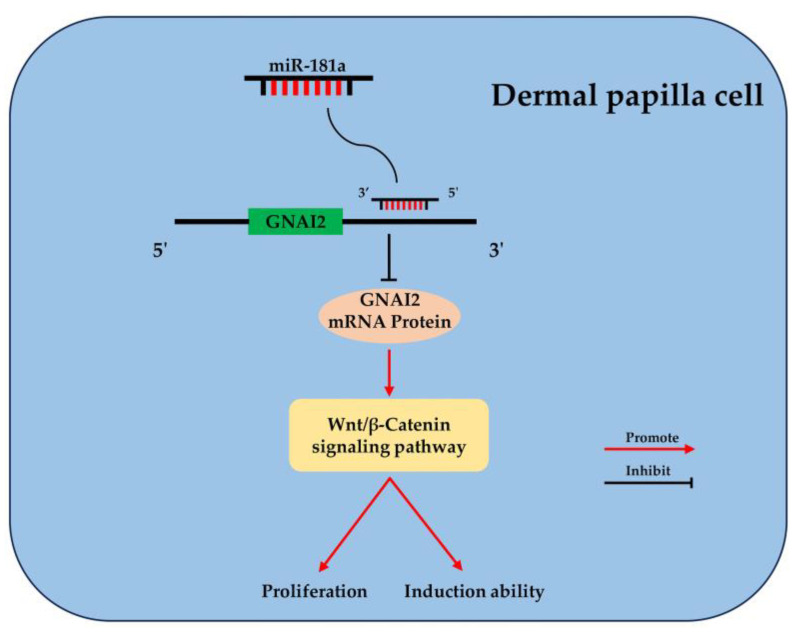
Schematic of miR-181a regulating the proliferation and induction ability of DPCs by GNAI2-Wnt/β-catenin signaling-pathway axis.

In conclusion, we revealed that miR-181a plays a negative role in the proliferation and induction ability of ovine DPCs by targeting *GNAI2* through the Wnt/β-catenin signaling pathway (Figure 7). Our study will provide a preliminary understanding of the involvement of non-coding RNA in the growth and development of sheep hair follicles.

## Figures and Tables

**Table 1:** MiRNA primer sequences used for qRT-PCR.

Gene	Primer Sequence (5′–3′)	Annealing Temperature (°C)
miR-181a	F: CGAACATTCAACGCTGTCG	58
	R: AGTGCAGGGTCCGAGGTATT	
Stem-loop primer	AACATTCAACGCTGTCGGTGAGTGTCGTATCCAG	60
	TGCGAATACCTCGGACCCTGCACTGGATACGAC	
*U6*	F: CTCGCTTCGGCAGCACA	60
	R: AACGCTTCACGAATTTGCGT	

**Table 2:** Gene primer sequences used for qRT-PCR.

Gene	Primer Sequence (5′–3′)	Product Size (bp)	Annealing Temperature (°C)	Accession Number
*GNAI2*	F: GAGTACCAGCTCAATGACTCTGCC	150	60	NM_001162566.1
	R: TAGGTCTTTGAAGGTGAAGTGCGT			
*PCNA*	F: CGAGGGCTTCGACACTTAC	97	60	XM_004014340.5
	R: GTCTTCATTGCCAGCACATT			
*CDK2*	F: AGAAGTGGCTGCATCACAAG	92	60	NM_001142509.1
	R: TCTCAGAATCTCCAGGGAATAG			
*IGF1*	F: TGTGCTTGCTCGCCTTCA	216	60	XM_027965760.2
	R: AGTACATCTCCAGCCTCCTCA			
*FGF7*	F: CTGCCAAGTTTGCTCTAC	286	60	NM_001009235.2
	R: CAGCCACTGTCCTGATTT			
*Versican*	F: TACAAAGGGAGGGTGTCGGT	226	60	XM_004009067.5
	R: AAGCCTTCTGTGCCATCTCA			
*CTNNB1*	F: GAGGACAAGCCACAGGATTAT	101	60	NM_001308590.1
	R: CCAAGATCAGCGGTCTCATT			
*TCF4*	F: AACCCTTTCGCCCACCAA	299	60	XM_012103768.4
	R: CAGGCTGATTCATCCCAC			
*LEF1*	F: CAGGTGGTGTTGGACAGATAA	179	60	XM_042251146.1
	R: ATGAGGGATGCCAGTTGTG			
*c-MYC*	F: CCCTACCCGCTCAACGACA	295	60	NM_001009426.1
	R: GGCTGTGAGGAGGTTTGC			
*cyclinD1*	F: CCGAGGAGAACAAGCAGATC	91	60	XM_027959928.2
	R: GAGGGTGGGTTGGAAATG			
*GAPDH*	F: TCTCAAGGGCATTCTAGGCTAC	151	60	NM_001190390.1
	R: GCCGAATTCATTGTCGTACCAG			

**Table 3:** Primers used for vector construction.

Primer Name	Primer Sequence (5′–3′)	Product Size (bp)	Annealing Temperature (°C)
OE-GNAI2	F: CTAGCGTTTAAACTTAAGCTT ATGCAGAGATCGCCGCTCG	1068	62
	R: CCACACTGGACTAGTGGATCCCTATCCAGAGATGCAGGCGCTG		
GNAI2-3′UTR-WT	F: AAAAGATCCTTTATT AAGCTT ACTGCAAACCTAGAAAACTTTTTAGAAA	242	60
	R: CATAGGCCGGCATAG ACGCGT CCAGGGCCACTGGGGTGG		
GNAI2-3′UTR-MT	F: TGCCTGACTGACGTCTACGTGTTTACACCCATCCC	6686	62
	R: TAGACGTCAGTCAGGCACGGAATCACCGGAAA		

**Table 4:** Sequences of oligonucleotide.

Fragment Name	Sequence (5′–3′)
miR-181a mimic	AACAUUCAACGCUGUCGGUGAGU
	ACUCACCGACAGCGUUGAAUGUU
mimic-NC	UUCUCCGAACGUGUCACGUTT
	ACGUGACACGUUCGGAGAATT
miR-181a inhibitor	ACUCACCGACAGCGUUGAAUGUU
inhibitor-NC	CAGUACUUUUGUGUAGUACAA
siRNA-432	GCGGGAGUACCAGCUCAAUTT
	AUUGAGCUGGUACUCCCGCTT
siRNA-551	GCAUCGUGGAGACGCACUUTT
	AAGUGCGUCUCCACGAUGCTT
siRNA-740	GCAUCGUGGAGACGCACUUTT
	AAGUGCGUCUCCACGAUGCTT
siRNA-NC	UUCUCCGAACGUGUCACGUTT
	ACGUGACACGUUCGGAGAATT

## Data Availability

All the data of this study are presented in the manuscript.
